# Generating samples for association studies based on HapMap data

**DOI:** 10.1186/1471-2105-9-44

**Published:** 2008-01-24

**Authors:** Jing Li, Yixuan Chen

**Affiliations:** 1 Electrical Engineering and Computer Science Department, Case Western Reserve University, Cleveland, OH 44106, USA

## Abstract

**Background:**

With the completion of the HapMap project, a variety of computational algorithms and tools have been proposed for haplotype inference, tag SNP selection and genome-wide association studies. Simulated data are commonly used in evaluating these new developed approaches. In addition to simulations based on population models, empirical data generated by perturbing real data, has also been used because it may inherit specific properties from real data. However, there is no tool that is publicly available to generate large scale simulated variation data by taking into account knowledge from the HapMap project.

**Results:**

A computer program (*gs*) was developed to quickly generate a large number of samples based on real data that are useful for a variety of purposes, including evaluating methods for haplotype inference, tag SNP selection and association studies. Two approaches have been implemented to generate dense SNP haplotype/genotype data that share similar local *linkage disequilibrium *(LD) patterns as those in human populations. The first approach takes haplotype pairs from samples as inputs, and the second approach takes patterns of haplotype block structures as inputs. Both quantitative and qualitative traits have been incorporated in the program. Phenotypes are generated based on a disease model, or based on the effect of a quantitative trait nucleotide, both of which can be specified by users. In addition to single-locus disease models, two-locus disease models have also been implemented that can incorporate any degree of epistasis. Users are allowed to specify all nine parameters in a 3 × 3 penetrance table. For several commonly used two-locus disease models, the program can automatically calculate penetrances based on the population prevalence and marginal effects of a disease that users can conveniently specify.

**Conclusion:**

The program *gs *can effectively generate large scale genetic and phenotypic variation data that can be used for evaluating new developed approaches. It is freely available from the authors' web site at .

## Background

With the completion of the HapMap project [1], large-scale, high-density single-nucleotide polymorphism (SNP) markers and information on haplotype structure and frequencies become available. A variety of statistical approaches have been proposed for association studies using haplotypes [2,3] and more are expected for whole genome association studies. The utilities of such approaches are frequently very difficult to obtain through analytical analysis. Evaluations on those methods commonly rely on experiments based on simulations or empirical data. For example, one can generate a large number of samples based on population models such as the coalescent theory, and the program ms [4] is routinely used in the community. Thus statistical properties of new approaches can be investigated in a controlled manner as functions of population parameters such as recombination rates, mutation rates, population structure and migration rates. But simulations based on population genetics models may not be able to capture the true property of LD in human populations, due to their simplified assumptions. Conclusions based on such simulated data may be misleading or inaccurate in reality. Some researchers [2,3] have used their in-house tools to generate empirical data based on real data, in hopes that their empirical data could inherit major properties of human populations such as LD patterns. But each group may use its own models and tools, and comparisons on results from different groups are usually impossible in general. A public available program that can generate a large number of independent samples based on real human data can be of great use for evaluating new proposed approaches. We have implemented a program in C++, called *gs*, that can efficiently generate such samples based on real data. Two heuristic approaches have been implemented. One is based on phased haplotype pairs and the other is based on haplotype block structures. The program can also directly take data from the HapMap project as its inputs. Our experiments show that genotype data generated by both approaches observe similar local haplotype structures and LD patterns as those in the input data, and also keep a proper level of variety. Therefore, genotype data can be used in testing algorithms for tag SNP selection and haplotype inference.

Simulation data is always indispensable for evaluation purposes. The major goal of this tool is to allow users to generate large scale simulation data for association studies, including both fine mapping and genome wide association. Both quantitative and qualitative traits have been incorporated. To generate disease phenotypes, users can either specify a one-locus disease model or a two-locus disease model with or without epistasis. Many studies and growing evidences have revealed the importance of epistasis in the etiology of complex traits. More and more efforts have been made to detect epistatic interactions [5-7]. For example, Marchini et al. studied the feasibility and power of a full two-locus model in the context of genome wide association studies and compared its performance with a single locus based approach and a two-stage design [5]. Evans et al. compared the performance of four different search strategies (i.e., a single-locus search, an exhaustive two-locus search, and two, two-stage procedures) to detect interacted loci using two-locus models [6]. Luliana et al. compared two natural two-stage approaches: the conditional approach and the simultaneous approach [7]. But for all the above studies, simulations were based on genotypes only at the trait loci. Although it is hardly possible to simulate thousands of individuals for hundreds of thousands SNP markers for thousands times for genome wide association studies due to storage and time constraints, our program can embed the disease genotypes into genome regions that mimic genomic content in human populations. Associations with markers can be tested in a more realistic scenario.

## Implementation

The program has implemented two methods to generate haplotypes/genotypes, *i.e.*, the extension method and the block method. Both methods can be used to generate qualitative and quantitative phenotype data. We first introduce the two methods in the context of generating case-control data, and then briefly discuss how quantitative traits can be generated. The extension to two-locus models will then be discussed.

### Extension Method

The first model is an extension to the one used in [2] that takes phased haplotype pairs as its inputs. For example, one can use the haplotype results from the HapMap project as inputs, which can be downloaded from the HapMap website. Users first create a disease model by specifying the disease allele frequency (DAF) and the penetrance of each genotype. Alternatively, users can define a disease model using the population prevalence and genotype relative risks. A simple relationship exists between penetrance parameters and genotype relative risk parameters [8]. Therefore, in the following we discuss our procedure only using one set of parameters, the penetrance. The program first picks a SNP *t *from the input data, where one of its two alleles has the frequency approximately equal to the DAF specified in the parameter file. This allele is regarded as the high-risk variant. (Alternatively, users can specify a particular SNP as the disease susceptibility locus.) To generate the genotype at the disease locus for a case, it first calculates the conditional probability of each genotype (homozygous wild, heterozygous, and homozygous mutant) given the individual being affected based on equation 1.

(1) $$Pr(g_{i}|case)=\frac{Pr(g_{i})Pr(case|g_{i})}{\sum_{j}Pr(g_{j})Pr(case|g_{j})}$$

The actual genotype *g *will then be selected based on the conditional distribution. The genotype frequencies in the above formula can be obtained from allele frequencies under the assumption of Hardy-Weinberg equilibrium. The probability of being affected given a particular genotype (penetrance) is given by users as a parameter. To generate the haplotype pairs *h*_1 _and *h*_2 _for this affected individual, the program randomly selects two haplotypes *h*_3 _and *h*_4 _from the inputs with the genotype at the disease locus *t *as required (*i.e.*, (*h*_3 _[*t*], *h*_4 _[*t*]) = *g*, where *h*_*i *_[*t*] denotes the allele at the *t*^*th *^locus on haplotype *h*_*i*_). In their original paper [2], haplotype *h*_1 _will be given the same alleles as *h*_3 _from locus *t *- *l *to *t *+ *l*, where *l *is a parameter that can be specified by users. To extend *h*_1 _to the right for one more locus, it randomly selects another haplotype *h*_5 _that has the same alleles as *h*_1 _from locus *t *- *l *+ 1 to locus *t *+ *l*, and let *h*_1 _[*t *+ *l *+ 1] = *h*_5 _[*t *+ *l *+ 1]. By iterating the above process, one can extend *h*_1 _to the right and then to the left. We found that LD patterns from samples generated this way greatly depend on the parameter *l *(data not shown). Even for one particular data set, the extend of LD may vary substantially in different segments. A single *l *can not accommodate all the cases. We have extended the above method by introducing two parameters, *l*_*min *_and *l*_*max*_. The overlapped length for both the initial assignment and the extension of *h*_1 _will be stochastically determined by two values *l*_*l *_and *l*_*r *_(*l*_*min *_≤ *l*_*l*_, *l*_*r *_≤ *l*_*max*_), one for each direction. The values of *l*_*l *_and *l*_*r *_depend upon the strength of local LD. More specifically, *l*_*r *_is initialized as *l*_*min*_. The value of *l*_*r *_is increased by 1 if the LD measure D' between locus *t *+ *l*_*r *_and locus *t *+ *l*_*r *_+ 1 is greater than a uniformly distributed random number between 0 and 1. The process will terminate when the value of D' is smaller than such a random number or when *l*_*r *_= *l*_*max*_. The value of *l*_*l *_can be determined similarly to the left. The haplotype *h*_1 _will be given the same alleles as *h*_3 _from locus *t *- *l*_*l *_to *t *+ *l*_*r *_initially. At each extension step, the procedure is the same as in the original paper [2], but with differences in determining the length of the overlapped region. For example, to extend to the right for one more locus, suppose the current locus (the right most one) is *t*_1_. The leftmost locus *t*_2 _of the overlapped region is stochastically determined based on pairwise LD with the constraint that *l*_*min *_≤ *t*_1 _- *t*_2 _≤ *l*_*max*_. A haplotype that shares the same segment with *h*_1 _from *t*_2 _to *t*_1 _will be randomly selected and its allele at *t*_1 _+ 1 will be copied to *h*_1_. A detailed description of the above procedure can be found in the manual of the program. The haplotype *h*_2 _can be obtained similarly. The required number of cases can be generated by repeating the process. One can generate normal individuals using the same approach based on the genotype distribution conditional on the fact that the individuals are normal. By using two parameters, the method takes both long-range LD (up to *l*_*max*_) and short-range LD into considerations.

### Block Method

**Figure 1:**
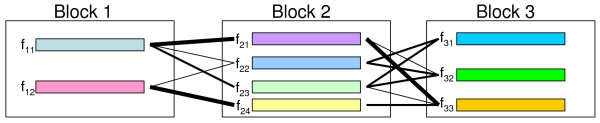
**Haplotype block structure as a Markov Model**. Each block is a state and each small rectangle within a block represents a common haplotype with its frequency denoted as *f*_*ij*_. The transition probabilities between adjacent blocks are depicted by lines with width representing quantity.

The second generating model is actually a Markov model based on haplotype block structures inferred from real data such as HapMap data. LD patterns such as a block-like structure have been commonly observed from experimental data for dense SNPs. Instead of directly using haplotype pairs, one can also take the haplotype block structures as inputs. As a Markov chain, each block is a state that consists of several common haplotypes controlled by an emission distribution. The connections of haplotypes between adjacent blocks are specified by a transition probability matrix. More specifically, for each block, the input data consist of the number of markers, the common haplotypes with their population frequencies, and the probabilities of each common haplotype connecting the common haplotypes in the next block. An example is given in Fig. 1. Such a structure can be inferred based on real data using some software such as Haploview [9]. To generate samples, users first specify a disease model (DAF and penetrances/relative risks) and the program selects a locus with its allele frequency approximately equal to the DAF. Users can also specify a disease locus directly. The genotype of a case (or a control) at the disease locus is generated in the same way as the extension method does. For each allele at the disease locus, a common haplotype with the allele embedded will be selected according to their frequency distribution. The two haplotypes will then be extended independently to both directions based on the transition probabilities. A large number of samples can be generated that will share similar LD patterns with real data but with different haplotypes and genotypes. To maintain a proper level of variety, we have considered SNPs that are not in any blocks, as well as possible rare haplotypes that do not exist from the input block file. For many block definitions, not all SNPs have to be within some blocks. To incorporate those missed SNPs, the original genotype data file that is used in generating the block structure has to be provided to the program. When generating a haplotype of a sample, the program imputes the missed SNPs sequentially based on their physical positions. For each position, an allele is chosen based on the allele in the previous position, and their frequencies and the pairwise LD between them estimated from the genotype data. Furthermore, rare haplotypes are usually been dropped in the Markov model. Only major haplotypes and their frequencies are available for each block. To incorporate rare haplotypes, we stochastically generates (only) one "rare" haplotype each time when a block is selected. The alleles of the haplotype are sampled solely based on the allele frequencies and pairwise LD. The frequency of the rare haplotype is defined so that the summation of haplotype frequencies within each block will be one (the summation of frequencies from common haplotypes only is often less than one from an input). The transition probabilities from the rare haplotype to haplotypes in the next block are proportional to the haplotype frequencies in the next block. When a block is selected again in generating another sample, a new rare haplotype will be generated and its frequency will be determined in a similar fashion. In such a way, every possible haplotype within a block will have a chance being selected as a rare haplotype for some samples. These new haplotypes will be rare overall in the samples because each time a different one might be selected. The procedure is designed so that it is slightly biased to haplotypes with common alleles.

### Quantitative Traits

To generate phenotypes for a quantitative trait, a quantitative trait nucleotide is chosen according to a specific allele frequency or a specific marker position provided by users. The phenotypic value of each individual is generated according to the classical single-locus quantitative trait model [10]. More specifically, users can specify the additive (*V*_*A*_) and dominance (*V*_*D*_) genetic variances attributable to the quantitative trait nucleotide as proportions of the total phenotypic variance. Denote the proportions as *π*_*A *_and *π*_*D*_. Let *V*_*O *_denote the variance due to all other (genetic and environmental) factors and assume its value is 1. Then *V*_*A *_and *V*_*D *_can be calculated based on

$$\begin{array}{ccc} \frac{V_{A}+V_{D}}{V_{A}+V_{D}+1}=\pi_{A}+\pi_{D}, & \text{and} & \frac{V_{A}}{V_{D}}=\frac{\pi_{A}}{\pi_{D}} \end{array}.$$

If one assumes that the phenotypic value of an individual can be partitioned as

(2) $$y=z+u_{i}=\{\begin{array}{ll} z & \text{homozygous wild}\\ z+(1+k)a & \text{heterozygous}\\ z+2a & \text{homozygous mutant} \end{array},$$

where *z *follows the standard normal distribution, *a *is half of the difference between two homozygous genotypic values (assume the mutant allele increases the phenotypic value), and *k *is a parameter representing the dominance effect, it is known [10] that *V*_*A *_and *V*_*D *_can be written as

(3) *V*_*A *_= 2*p*(1 - *p*)*a*^2^(1 - *k*(2*p *- 1))^2^, and *V*_*D *_= (2*p*(1 - *p*)*ak*)^2^,

where *p *is the frequency of the mutant allele. Thus *a *and *k *can be obtained based on the above equations. The phenotypic value of an individual can be calculated by substituting *a *and *k *into Equation 2.

### Two-Locus Models

For a one-locus diallelic disease model, users can specify at most three penetrances, one for each genotype. The number of parameters reduces to two if one considers some commonly used models such as dominant models, recessive models, or additive models. For a two-locus diallelic disease model, two interacting sites are involved, and in theory, one can specify nine penetrances, one for each genotype combination of the two sites. There are also many restricted models with less than 9 free parameters that are of great interest to the community. By focusing on fully penetrant models (the probability of an individual being affected is either 1 or 0 for any given genotype combination), Li et al. [11] enumerated all the 512 possible combinations and summarized 50 unique ones. We believe that it is necessary to allow incomplete penetrances (any values between 0 and 1) in simulating complex diseases. But with incomplete penetrances, the possible number of models becomes infinite. To incorporate both incomplete penetrances and some commonly used restrict models, the *gs *program provides two distinct methods to allow users to specify a two-locus model. For the first method, all the nine parameters are free and users have the freedom to assign each penetrance with any probability value. Thus the program can generate datasets with any desired disease models. However, in many cases, it may not be intuitive to assign values for the nine penetrances directly. Instead, users might have some information about the population prevalence of a particular disease, and information on marginal effects (such as genotype relative risks or genotype odds ratios) of individual locus. And they might want to test the power of their method under some particular two-locus interaction models. To meet such needs, the *gs *program has implemented nine commonly used models in the literature. For each of these nine models, users only need to specify the population prevalence *p*, and genotype odds ratio(s) 1 + *θ *for each locus. The program can automatically calculate the penetrance table. For example, Table 1 represents a jointly dominant-dominant model, where at least one disease allele is required at both loci to increase disease odds and both loci have the same effect size. Each cell of the table represents the odds of the disease for an individual with the corresponding genotype combination. Let *Pr*(*D*|*g*_*i*_) denote the probability of an individual being affected given its genotype combination of *g*_*i *_(*i.e.*, the penetrance of *g*_*i*_), and let *Pr*($\bar{D}$|*g*_*i*_) denote the probability of an individual not being affected given its genotype *g*_*i*_. Based on the definition of the odds of a disease

**Table 1:** Odds table for the jointly dominant-dominant model. *α *is the baseline odd and (1 + *θ*) is the genotype odd ratio.

	bb	Bb	BB
aa	*α*	*α*	*α*
Aa	*α*	*α*(1 + *θ*)	*α*(1 + *θ*)
AA	*α*	*α*(1 + *θ*)	*α*(1 + *θ*)

(4) $$ODD_{g_{i}}=\frac{Pr(D|g_{i})}{Pr(\bar{D}|g_{i})}=\frac{Pr(D|g_{i})}{1-Pr(D|g_{i})},$$

the penetrance of *g*_*i *_can be calculated using the following formula,

(5) $$Pr(D|g_{i})=\frac{ODD_{g_{i}}}{1+ODD_{g_{i}}}.$$

A corresponding penetrance table is give in Table 2. Once the population prevalence *p *and the genotype odds ratio (1 + *θ*) are fixed in this model, the baseline value *α*, which indicates the odds of disease when the two loci do not carry any disease alleles, can be calculated by plugging the terms in Table 2 into the following formula,

**Table 2:** Penetrance table for the jointly dominant-dominant model, where *α *and *θ *are defined in Table 1.

	bb	Bb	BB
aa	$\frac{\alpha }{1+\alpha }$	$\frac{\alpha }{1+\alpha }$	$\frac{\alpha }{1+\alpha }$
Aa	$\frac{\alpha }{1+\alpha }$	$\frac{\alpha (1+\theta )}{1+\alpha (1+\theta )}$	$\frac{\alpha (1+\theta )}{1+\alpha (1+\theta )}$
AA	$\frac{\alpha }{1+\alpha }$	$\frac{\alpha (1+\theta )}{1+\alpha (1+\theta )}$	$\frac{\alpha (1+\theta )}{1+\alpha (1+\theta )}$

(6) $$p=Pr(D)=\sum_{i}Pr(D|g_{i})Pr(g_{i}).$$

The frequencies on genotype combinations (*Pr*(*g*_*i*_)) can be obtained from allele frequencies under the Hardy-Weinberg Equilibrium assumption. The details of all the nine built-in models can be found in the manual of the program. Once the three by three penetrance table is ready, the *gs *program can calculate the conditional probability of each genotype combination given affected status using a similar formula as Equation 1. Actual genotypes at the two loci will be selected based on the conditional distribution. The haplotypes will then be selected independently for these two loci, using either the extension method or the block method. The above two-locus model does not explicitly consider linkage disequilibrium between them (*i.e.*, the two loci are assumed to be in linkage equilibrium). We will consider models with two disease loci that are in LD, as well as haplotype-based disease models in a subsequent version.

### Formats

The inputs to the program can be obtained from HapMap project or from users' own research projects. Phased haplotype pairs from HapMap website can be directly incorporated into the software using the extension method. To use the block method, one can generate block structures from phased haplotype pairs using the program Haploview [9]. Both approaches allow us to generate large data sets that mimic the true local LD from human populations. A variety of parameters can be specified by users, including some that control the output formats. There are basically three different output formats including a widely used format in the community, linkage format. Users can choose to output phased haplotype data or unphased genotype data. The disease causing SNP can be kept or removed in the final outputs. More details about file formats can be found in the manual of the program. The program does not directly model population structures. But one can create a data set that is a mixture of different populations with different allele frequencies and effects by combining samples generated based on inputs from different populations. To generate random numbers, the Mersenne Twister algorithm [12] has been adopted.

## Results and Discussion

### Datasets

We have tested both generating methods using two different data sets. The first dataset consists of all 10 ENCODE regions from the HapMap project, which is a portion of the 44 regions from the ENCODE project. These ENCODE regions were selected either manually, or randomly based on gene density and level of non-exonic conservation. Details about selection criteria can be found at ENCODE website [13]. The 10 HapMap-ENCODE regions were resequenced in 48 unrelated individuals and genotype data were obtained from about 20 thousand SNPs of all 270 HapMap samples. The haplotype pairs of each individual can be downloaded directly from the HapMap phase I data release. For each region, we take the parental haplotypes from 30 trios in a population with northern and western European ancestry. We mainly present our results using one region (ENr112 on chromosome 2p16.3) in the paper and results on other regions can be found on our website as supplementary materials. The second dataset is from a genome wide association study of a neurological disease [14]. This dataset is one of the first sets of publicly available genome wide SNP data, which can be downloaded from Coriell Institute for Medical Research [15]. In its first stage, the study genotyped 550k SNPs across genome using the Illumina Infinium II SNP chip, for 276 patients with sporadic amyotrophic lateral sclerosis (ALS) disease and 271 normal individuals. We have to preprocess the data before running our program, by either inferring haplotypes or predicting haplotype block structures. We chose the most recent version of a widely used program fastPhase [16] for haplotype inference. Haploview was selected for haplotype block structure prediction.

Because the phenotypic models are standard, we mainly assess the performance of our program in terms of the similarity of haplotype structures between simulated data and input data. The haplotype structure is measured mainly by two quantities, *i.e.*, the number of haplotype blocks and the average number of block length within each region, as well as visual examination of local LD patterns. For each region, its haplotype structure is first inferred using Gabriel's method [17] implemented in Haploview based on its default values, and the number of haplotype blocks and the average number of block length is obtained. For each method and for each fixed set of parameters, 100 replicates will be generated and these simulated data will be reloaded to Haploview to obtain their haplotype structures. The average number of blocks and average length of blocks of the 100 replicates will be compared to values obtained from the input data.

### Parameters

**Figure 2:**
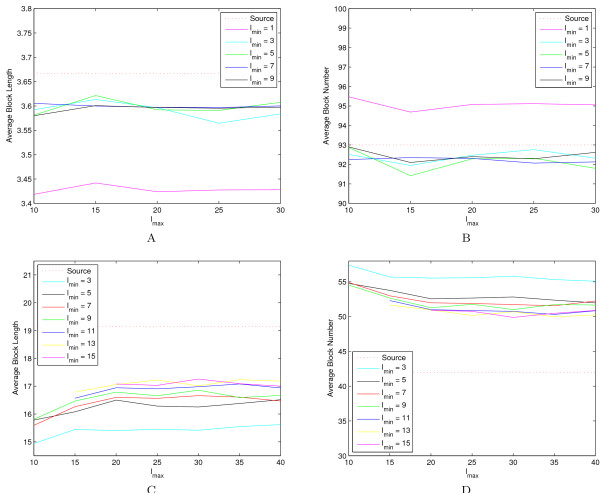
**Effects of *l*_*min *_and *l*_*max *_on block structures for the extension method**. The average length of blocks (A) and the average number of blocks (B) using different combinations of *l*_*min *_and *l*_*max *_on the ALS dataset. Panels C and D illustrate the two measures on region ENr112.

For the extension method, there are two important parameters *l*_*min *_and *l*_*max*_, which are used to adjust the segment length in the extension method based on local LD values. To evaluate the effect of *l*_*min *_and *l*_*max *_on haplotype structures, we take one region from the ALS dataset and one ENCODE region (ENr112 on chromosome 2p16.3), and perform extensive tests using different combinations of *l*_*min *_and *l*_*max*_. The ALS region (3.3 Mb) was randomly selected from chromosome one with 500 SNPs. The ENr112 region (500 kb) has 1157 SNPs. The major difference between these two regions is SNP density. Because ENr112 consist of a much dense SNP set, it has a much larger average block length (19 SNPs) comparing with the ALS data (3.7 SNPs). We tested a wide variety of combinations of these two parameters on the ALS data (*l*_*min *_= 1, 3, 5, 7, 9; *l*_*max *_= 10, 15, 25, 30) and the ENr112 region (*l*_*min *_= 3, 5, 7, 9, 11, 13, 15; *l*_*max *_= 10, 15, 25, 30, 35, 40). Results (Figure 2) show that the performance of the program is quite robust and consistent. The program shows suboptimal results only when *l*_*min *_takes extremely small values (*e.g.*, 1 for ALS and 3 for ENr112). This result demonstrates that the program gains the flexibility to capture local LD by using these two parameters. The optimal choice of the two parameters mainly depend on marker densities. But for each density, a variety of values will work almost equally well. For our experiments, we use (*l*_*min*_, *l*_*max*_) = (3, 9) for the ALS data (2 SNPs/10 kb on average) and (*l*_*min*_, *l*_*max*_) = (9, 30) for ENCODE regions (2–4 SNPs/kb on average).

### Allele frequencies, LD and block structure

**Figure 3:**
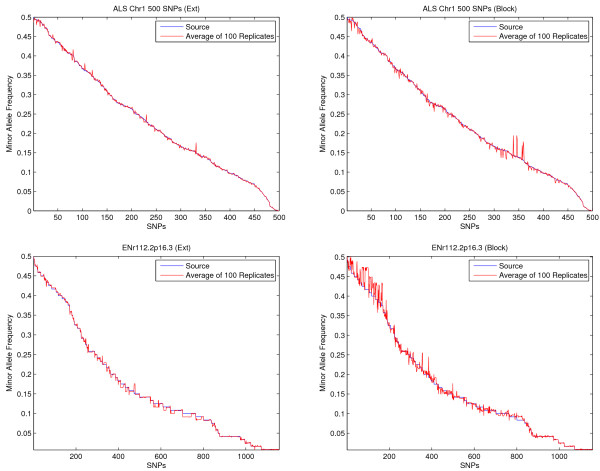
**Allele frequencies from simulated data**. Comparison of the average allele frequencies from 100 replicates (red) and the allele frequencies from the original input data (blue) based on the extension method (left) and the block method (right) using the ALS data (top) and HapMap region ENr112 (bottom). For each panel, the SNPs in the original data are aligned on the *x *axis in the decreasing order of their minor allele frequencies. The *y *axis is the measure of allele frequencies.

**Figure 4:**
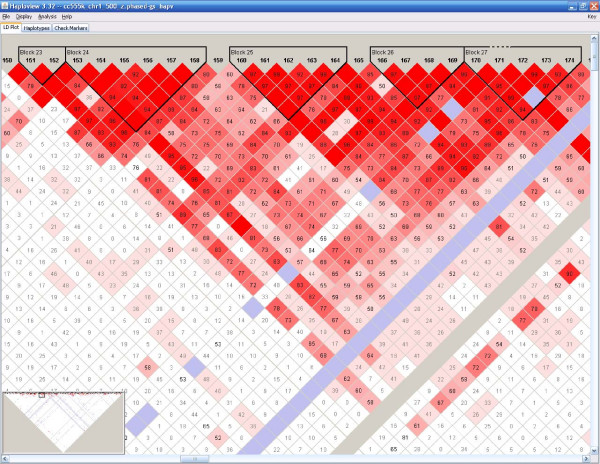
**Haplotype block structure from original input data (ALS)**. The structure was generated using Haploview program. Each diamond and its color represent the strength of pairwise LD with the two SNPs on its diagonal lines. Detail explanations can be found from its website at .

**Figure 5:**
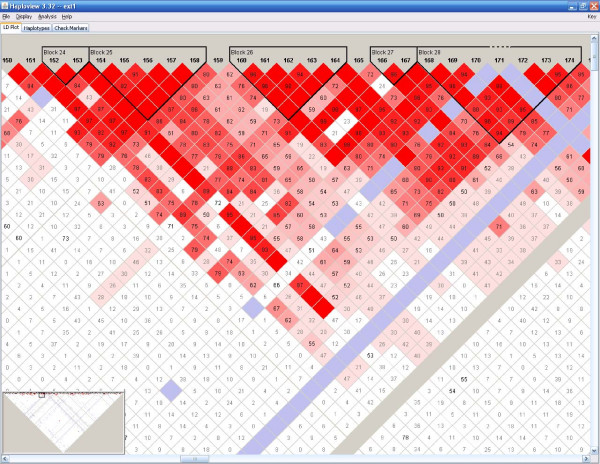
Haplotype block structure from one replicate generated by the extension method (ALS).

We first compare allele frequencies of simulated data and original input data for both approaches on 11 regions (ALS plus 10 ENCODE regions). Results show that allele frequencies from simulated data are very close to those from original data for both methods (except for the block method on region ENr113.4q26 for some unknown reasons). Figure 3 shows the results of the two methods on the ALS region and the ENr112 region. SNPs in the input data are arranged along the *x *axis in a decreasing order according to their minor allele frequencies. The original frequency is represented by a blue line and the average frequency from 100 replicates in simulated data is represented by a red line. Only small variations can be observed for both datasets and for both approaches. Overall, the block method seems to have higher variation than the extension method. In terms of haplotype structures, data generated from both methods have shown a "blocky" structure (an example is given in Fig. 4, 5, 6 from the ALS region). Local LD values within blocks are very similar to those in the original human data. Simulated data has also shown some varieties as expected. The results from all 11 regions from 100 replicates for both methods are summarized in Table 3. The similarity of simulated data and input data is evaluated in terms of block parameters such as the number of blocks, the average number of markers in each block, the percentage of overlapped markers within blocks. In general, the extension method shows a consistent performance across the regions tested in all measures. The block method performs well on the ALS data, and the percentages of overlapped SNPs in original data and simulated data are high. However, the block method generates many more blocks with smaller sizes in simulated data. We suspect that the performance of the block method greatly depends on SNP density, and investigate the dependence using additional experiments. We take the same physical region ENr112 from chromosome 2p16.3, but construct three different densities: HapMap phase I data, phase I with redundant SNPs removed (a SNP is redundant if it can be determined completely by another SNP in the samples), ALS data. The number of blocks and the average length of blocks in the original data reduced from 42 to 25, and from 19 to 5, respectively, when the number of SNPs reduced from 1157 to 165. Those numbers in simulated data also reduced from 67 to 23, and from 12 to 5, respectively (Table 4, first three rows). The block method tends to generate more blocks with small sizes when the input data consist of extremely high dense SNPs because small variations will break long blocks (with dozens of SNPs) into smaller ones. In addition, for blocks with large number of SNPs, the summation of all common haplotype frequencies is usually far less than one, which leads to a higher chance of introducing artificial rare haplotypes and breaks long blocks into short ones. We further investigate how the incorporation of pairwise LD when imputing rare haplotypes and SNPs that are not within any blocks affects block structures. The last row in Table 4 presents the result of the block method without using pairwise LD. Comparing with row 2, not considering pairwise LD when imputing missing will result in smaller number of longer blocks. Further examinations reveal that small blocks (*e.g.*, with two SNPs) can be formed in the imputed regions when using pairwise LD.

**Figure 6:**
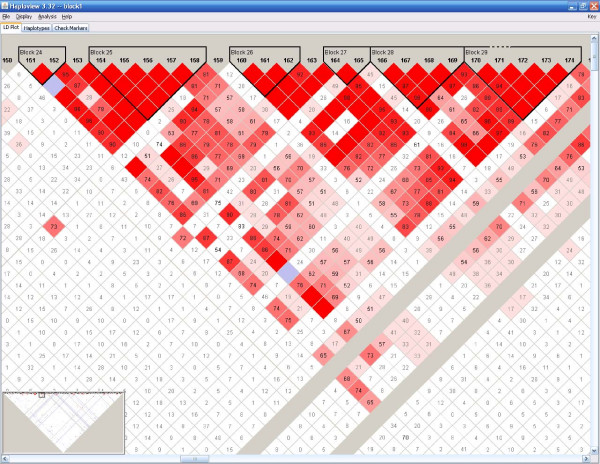
Haplotype block structure from one replicate generated by the block method (ALS).

**Table 3:** Comparison of block structures of simulated data and original data.

Dataset	Original		Extension			Block		
	
	# blocks	avg len	# blocks	avg len	overlap	# blocks	avg len	overlap
ALS Chr1 500 SNPs	93	3.67	91.95	3.61	91.98%	92.91	3.54	94.34%
ENr112.2p16.3	42	19.14	51.02	16.86	98.34%	67.30	12.21	97.17%
ENr131.2q37.1	54	16.85	54.95	17.02	96.88%	79.59	11.21	95.26%
ENr113.4q26	23	44.78	26.55	39.96	98.11%	91.51	10.78	93.87%
ENm010.7p15.2	28	15.36	27.47	16.05	96.98%	31.60	13.68	96.99%
ENm013.7q21.13	22	33.05	17.59	41.58	96.27%	57.58	11.88	91.08%
ENm014.7q31.33	23	37.70	19.15	45.33	96.79%	70.3	11.69	92.04%
ENr321.8q24.11	24	21.50	26.12	19.88	97.08%	32.01	15.87	96.38%
ENr232.9q34.11	24	17.29	31.08	13.85	96.85%	36.02	11.56	96.94%
ENr123.12q12	31	21.81	26.57	27.02	97.27%	48.40	13.92	96.15%
ENr213.18q12.1	19	29.74	22.32	26.40	97.68%	36.83	15.41	96.71%

**Table 4:** Results of the block method on the same physical region with different densities and different missing data imputing methods.

Dataset	Original			Block		
	
	# SNPs	# blocks	avg len	# blocks	avg len	overlap
Phase I	1157	42	19.14	67.3	12.21	97.2%
Phase I (nr)	441	38	6.29	53.2	4.85	96.2%
ALS	165	25	4.92	23.2	5.2	94.4%
Phase I (nr, nLD)	441	38	6.29	30.8	7.25	71.4%

### Efficiency

The *gs *program is efficient and runs fast. It can generate 100 replicates of 200 individuals with around 500 markers within minutes on a desktop with a 3.4 GHz processor and 2 GB memory. We have also tested the program in the context of genome wide association studies using the ALS data, which is based on Illumina 550K SNP chips [14]. For the extension method, the *gs *program needs haplotypes as its inputs. The program fastPhase [16] was used to obtain haplotype information from genotypes. We randomly selected one chromosome (chromosome 6) with 36,381 SNPs, and it took fastPhase about 96 hours to obtain the haplotype pairs of 271 (normal) samples from the ALS dataset. This limits us to perform the tests using only one chromosome for the extension method in this study. On average (from five replicates), it took about 140 minutes in generating 1000 cases and 1000 controls from the haplotype pairs of the 271 samples for the 36K SNPs. Because our algorithm is linear in the number of SNPs, the estimated time to generate 550K SNPs in a sequential manner is roughly 33 hours. If a cluster with multiple processors were used, the computation for different chromosomes can be run independently, and the total time is bounded by the running time of the chromosome with the largest number of SNPs. For the block method, *gs *needs haplotype block structures as inputs. However, Haploview cannot handle datasets with such sizes at a chromosome level for structure prediction. We could not test the block method at the genome level. Based on our experiences on small datasets, the block method is slight faster than the extension method. Therefore, for simulations at a genome level, the block method may require less or similar time as the extension method does.

## Discussion

We develop a program to generate genotypes/haplotypes by perturbing real data based on two approaches. Qualitative or quantitative phenotypes can be generated based on genetic models. The goal of generating simulated data by perturbation is to create a large number of replicates that share similar properties with real data. For the extension method, the randomness is mainly from the sampling procedure at each step when an extension occurs. Experiments show it is robust across a wide range of parameter values and SNP densities. For the block method, noise can be introduced when imputing rare haplotypes or imputing SNPs not within original blocks, in addition to deviations due to random sampling. Test results show it is more suitable for data with small blocks (length ≤ 10, e.g., Illumina 550K SNP chips). One should also notice one practical limitation while simulating data using perturbations. When simulated data only inherit properties from the set of input samples, it may never be able to represent the whole population if the inputs are biased. For example, the HapMap project only consists of a small number of samples in each of its ethnic groups. Rare SNPs may not be typed and rare haplotypes may not be observed. Data generated based on HapMap samples cannot reveal the true distribution of rare SNPs. This problem will be alleviated with the availability of more real data in the future.

The standard error of the average block length of each ENCODE region is usually great (data not shown), which reflects the fact that block lengthes vary dramatically. Therefore, the number of blocks and the average length of blocks can not give a whole picture of the block structure. Visual examination reveals that local LD structures from simulated data generated by both methods show high concordance with those from original data. However, none of the two methods can have a good control on long range LD. Another limitation of the program is that it requires inputs to be either haplotypes or haplotype structures. Both of them have to be inferred from genotype data, and the inference usually takes much longer time than the simulation itself. We will investigate new perturbation approaches directly based on genotype data. The method in generating genotypes at the disease loci assumes that the alleles are in Hardy-Weinberg equilibrium (HWE) in the population where samples are drawn. It is known that a population can deviate from the HWE due to many reasons [18]. For example, if there are differences in the survival rates of individuals with different genotypes, deviations from HWE might occur. Some other reasons that cause deviation from the HWE include non-random mating, preferential selection of samples, etc. The samples generated by our program are not suitable to study diseases that might deviate from HWE for those reasons.

## Conclusion

We have developed a software tool (*gs*) that can efficiently generate a large number of samples with genomic and phenotypic variations based on HapMap data or any real data. Experiments show that the two approaches can produce data that share similar local LD patterns as those in the input data. Both single-locus and two-locus disease models have been incorporated in the implementation. The data generated by the program can be used for a variety of purposes, including the evaluation of algorithms for haplotype inference, tag SNP selection and association studies. It can be used to evaluate algorithms for gene fine mapping as well as algorithms for genome wide association studies.

## Availability and requirements

Project name: Generating Samples based on HapMap data

Project home page: 

Operating system(s): Windows and Linux/Unix.

Language: C++.

Any restrictions to use by non-academics: none.

## List of Abbreviations

SNP – single-nucleotide polymorphism;

LD – linkage disequilibrium;

DAF – Disease allele frequency;

## Authors' contributions

JL initiated and designed the research, and wrote the manuscript. YC implemented the program and performed the tests. Both authors have read and approved the final version of the manuscript.
